# Comparison of remineralization ability of tricalcium silicate and of glass ionomer cement on residual dentin: an in vitro study

**DOI:** 10.1186/s12903-024-04475-4

**Published:** 2024-06-26

**Authors:** Elif Kuru, Nesrin Eronat, Murat Türkün, Dilşah Çoğulu

**Affiliations:** 1 Department of Pediatric Dentistry, Usak University School of Dentistry, Cumhuriyet, Merkez/Usak, 64200 Turkey; 2 Department of Pediatric Dentistry, Ege University School of Dentistry, Erzene, Bornova/İzmir, 35040 Turkey; 3 Department of Restorative Dentistry, Ege University School of Dentistry, Erzene, Bornova / İzmir, 35040 Turkey

**Keywords:** Bioactive dental materials, Calcium silicate cement, Dentin remineralization, Glass ionomer cement

## Abstract

**Objective:**

This study aimed to compare the remineralization effects of a calcium silicate-based cement (Biodentine) and of a glass ionomer cement (GIC: Fuji IX) on artificially demineralized dentin.

**Methods:**

Four standard cavities were prepared in dentin discs prepared from 34 extracted sound human third molars. In each disc, one cavity was covered with an acid-resistant varnish before demineralization (Group 1). The specimens were soaked in a chemical demineralization solution for 96 h to induce artificial carious lesions. Thereafter, one cavity each was filled with Biodentine (Group 2) and GIC (Group 3), respectively, and one carious lesion was left unrestored as a negative control (Group 4). Next, specimens were immersed in simulated body fluid (SBF) for 21 days. After cross-sectioning the specimens, the Ca/P ratio was calculated in each specimen by using scanning electron microscopy–energy-dispersive X-ray spectroscopy (SEM-EDX). Finally, data were analyzed using repeated-measures ANOVA with post-hoc Bonferroni correction.

**Results:**

Both cement types induced dentin remineralization as compared to Group 4. The Ca/P ratio was significantly higher in Group 2 than in Group 3 (*p* < 0.05).

**Conclusion:**

The dentin lesion remineralization capability of Biodentine is higher than that of GIC, suggesting the usefulness of the former as a bioactive dentin replacement material.

**Clinical relevance:**

Biodentine has a higher remineralization ability than that of GIC for carious dentin, and its interfacial properties make it a promising bioactive dentin restorative material.

## Introduction

Management of deep dentin caries is a common challenge encountered in daily practice. Complete caries removal to reach sound dentin is debated, due to concerns about potential complications, such as pulp exposure, toothache, and weakening of the tooth structure [1]. Current caries management approaches focus on the removal of soft and infected dentin, after which the caries-affected dentin (CAD) is permanently sealed [2]. Because of the lower bond strength [3], increased water content [4], and lower strength [5] of the demineralized dentin, remineralization of CAD is a key factor for long-term survival [6].

Bioactive dental materials have been developed to induce dentin remineralization, strengthen the restoration–dentin interface, and make dental hard tissues resistant to demineralization. A dental restorative material is considered “bioactive” if it can actively stimulate or direct specific cellular and tissue responses, in addition to its primary function of restoring or replacing missing tooth structure [7]. Various approaches have led to the development of different bioactive materials for diverse applications on tooth surfaces. Some bioactive particles are incorporated into resin composites and bonding agents to induce remineralization in enamel and cavosurface margins, preventing bacterial leakage and secondary caries. These particles include fluoride-releasing fillers, quaternary ammonium silica dioxide nanoparticles, bioactive glass, hydroxyapatite, amorphous calcium phosphates, nano-calcium phosphates, and mono-, di-, and tetracalcium phosphates [8–12]. Additionally, ion-releasing glass ionomer cements (GICs) and calcium-silicate cements are used to induce dentin remineralization in deep cavities and (CSCs) are used as pulp capping materials to preserve tooth vitality [13, 14].

Glass ionomer cements (GICs) are water-based restorative materials known for their remineralization ability. Conventional GICs are available in powder–liquid form; the powder consists of fluoro-aluminosilicate glass particles, whereas the liquid is an aqueous solution of polyalkenoic acid (i.e., polyacrylic acid) [15]. Recently, strontium has been added to commercially available GICs, such as GC Fuji IX [13]. GICs set via an acid–base reaction between the ion-leachable glass particles and the polyalkenoic acid. When freshly mixed GIC is applied to dentin, ion exchange occurs. Subsequently, fluoride and calcium/strontium leach out of the cement, whereas calcium and phosphate ions move out of the dentin because of the acidic environment [16]. This release of fluoride and calcium/strontium plays a role in apatite formation, providing GICs with the ability to remineralize carious dentin. Thus, GICs are widely used for minimally invasive dentin restorations [17, 18].

Calcium silicate cement (CSC) is a water-soluble, alkaline bioactive material that contains tri-calcium silicate [14]. Since its introduction in dentistry, CSC has mostly been used for endodontic applications, due to its extended setting time and discoloration [19]. Additionally, it is used as a bioactive material because of its ability to induce apatite formation by releasing Ca^2+^ and OH^−^ ions [19]. Biodentine™ is a type of CSC that was introduced as a “dentin replacement material” [20]. Its shorter setting time distinguishes it from the other CSCs proposed for use as coronal restoration materials. Recent studies have demonstrated the remineralization capability of Biodentine [21–24]. Hence, Biodentine can be used for the management of deep dentin caries to induce CAD remineralization and preserve pulp vitality.

Biodentine has been proposed as a potential alternative to GIC for lining and/or pulp capping procedures involving CAD. However, to our knowledge, current literature indicates that Biodentine is primarily used for pulp capping or endodontic purposes, with limited research on its application as a dentin restorative material in minimally invasive treatments such as selective caries removal [25–28]. To be useful as a restorative material in CAD, Biodentine must induce remineralization as effectively as GIC. However, few studies have compared the remineralization abilities of CSC and GIC on carious dentin lesions.

This study thus evaluated the remineralization ability of calcium silicate-based Biodentine and GIC (Fuji IX) on artificial dentin lesions using scanning electron microscopy (SEM) and energy-dispersive X-ray analysis (EDX). The null hypothesis was that the potential to remineralize artificially induced carious dentin would not be significantly different between the two cement types.

## Methods and materials

### Study methods

**Fig. 1:**
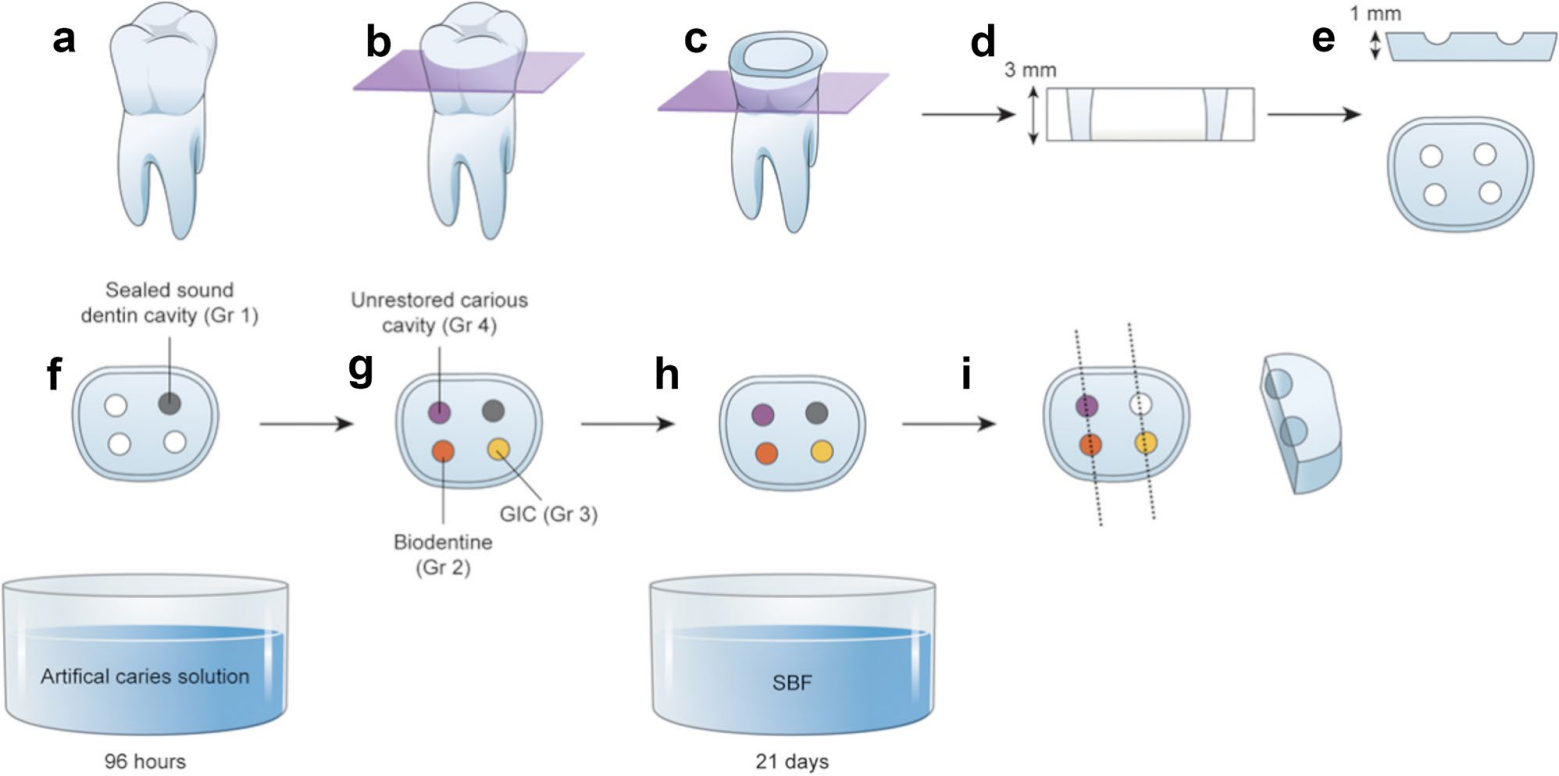
Study design and methodology. (**a**) Non-carious permanent molars were selected. (**b**) The occlusal third of the crown was removed. (**c**) Roots and the cervical third of the tooth were removed. (**d**) Next, 3-mm-thick dentin slices were obtained. (**e**) Four standardized, round dentin cavities with a depth of 1 mm were prepared. (**f**) Hard wax was used to seal one of the four disc cavities, which was used as a positive control (Group 1). The specimens were soaked in artificial caries solution for 96 h. (**g**) One of the carious lesions in each sample was left unrestored and was used as a negative control (Group 4). The other two carious lesions were restored using either Biodentine (Group 2) or glass ionomer cement (GIC; Group 3). (**h**) The specimens were soaked in the remineralization solution (simulated body fluid [SBF]) for 21 days. (**i**) The specimens were sectioned in the buccolingual direction, through the centers of the restorations, and scanning electron microscopy–energy-dispersive X-ray spectroscopy (SEM-EDX) analysis was performed

A modified version of the method described by Pires et al. was used to prepare the specimens (Fig. 1) [29]. To achieve standardization, four cavities (one for each group) were prepared on a single dentin disc. The cavities were randomly divided into four groups: positive control, negative control, GIC, and Biodentine.

Sample size was calculated using the G*Power software (version 3.1.9.2 for Macintosh, Heinrich-Heine, Duesseldorf, Germany). Given the lack of similar studies, the minimum sample size required to compare the four dependent groups was calculated. A minimum sample size of 136 cavities (in 34 teeth) was required, assuming an alpha error level of 0.05, power of 0.80 (moderate), and an effect size of 0.25 [30].

### Tooth selection and sample preparation

This study was approved by the Medical Ethics Committee of Ege University (19-4T/31). Non-carious third molars (extraction indicated due to tooth impaction) were obtained from patients treated at Department of Oral Surgery of Ege University, who provided informed consent. Soft tissues were removed immediately after extraction by cleaning the teeth with water and pumice. Teeth with cavities, fractures, or discolorations were excluded. Selected teeth were randomly assigned a number (1–34) and stored in a 0.1% thymol aqueous solution at 4 °C until used (within 1 month).

Dentin slices (3-mm-thick) were obtained using a 0.3-mm-thick diamond disc (IsoMet Blade 5LC, Buehler Inc, Lake Bluff, IL, USA) mounted on a cutting machine (Fig. 1a–d). Four regular round-shaped dentin cavities (2 mm diameter × 1 mm depth) were prepared in each specimen using a spherical diamond bur and high-speed dental handpiece with water-cooling (Fig. 1e). An acid-resistant nail polish was applied to all surfaces, excluding the cavities.

### Induction of artificial caries

The specimens were soaked in demineralization solution (pH 5.3, 37 °C) containing 50 mM acetic acid, 3 mM CaCl_2_ × H_2_O, 3 mM KH_2_PO_2_, and 6 mM methyl-hydroxydiphosphonate [31] for 96 h to induce artificial caries lesions. The pH of the solution was checked daily and adjusted if necessary, using 10% HCl or 10 M KOH. All chemicals were purchased from Sigma-Aldrich (St. Louis, MO, USA). The solution was prepared 1 week before starting the experiments. The sample-storage solution was renewed daily to maintain continuous ion exchange.

**Fig. 2:**
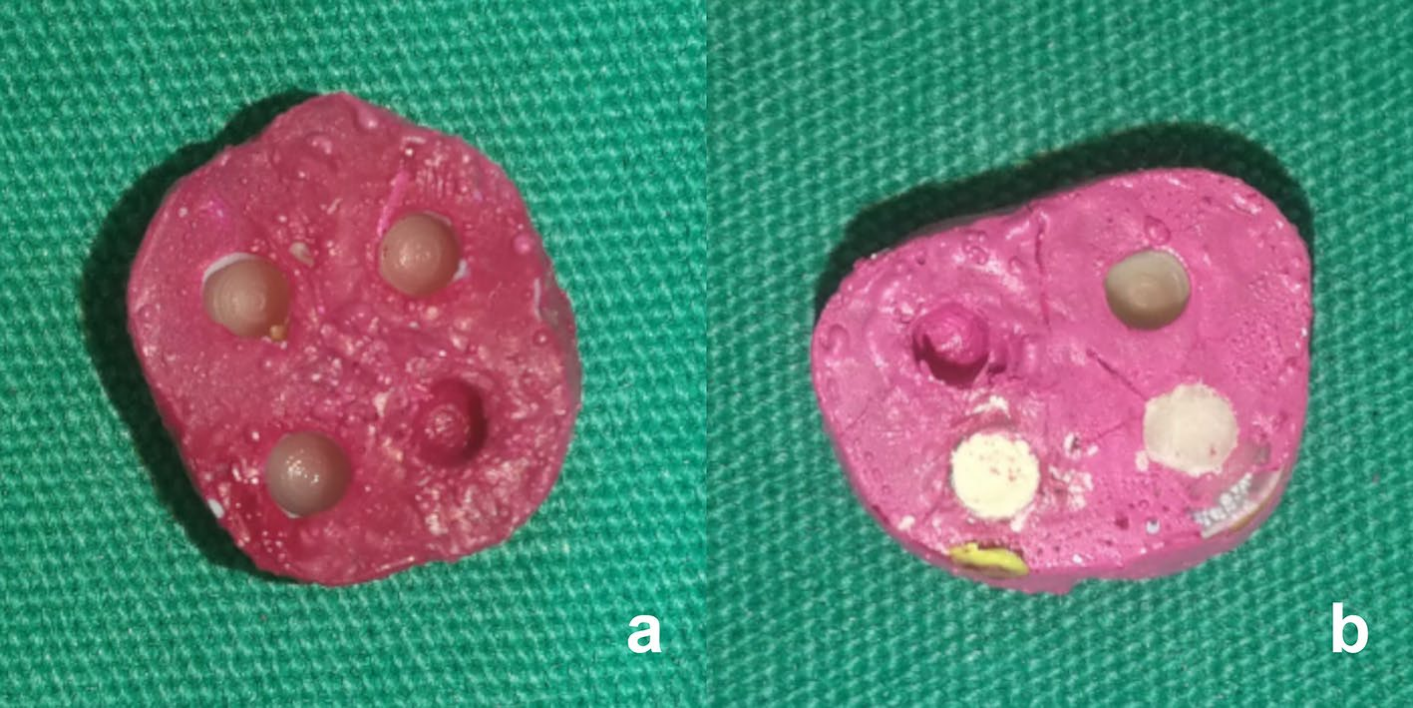
Gross view of the specimens before and after application of restorative materials. (**a**) Carious cavities after the demineralization process. (**b**) The application of restorative materials

After demineralization, the cavities were rinsed with distilled water and air dried (Fig. 2a). Acid-resistant varnish and hard wax were used to seal one of the four cavities on each disc, which was defined as the positive control (Group 1) (Fig. 1f). Two carious cavities were restored using either Biodentine (Septodont, Saint Maur des Fosses, France) (Group 2) or GIC (Fuji IX, GC Europe NV, Leuven, Belgium) (Group 3), as shown in Figs. 1g and 2b. The remaining carious cavity in each sample was assigned to the negative control group (Group 4) and was left unrestored. All materials were prepared according to the manufacturer’s instructions and applied to the cavities immediately after mixing.

### Preparing the remineralization solution

Simulated body fluid (SBF; pH 7.4, 37 °C) was prepared according to the method proposed by Kokubo and Takadama [32]. The chemical composition of the SBF is presented in Table 1. The pH of the SBF was adjusted using an HCl solution. The dentin samples were stored in the SBF for 21 days at 37 °C; the SBF was replaced daily.

**Table 1:** Chemical composition of Simulated Body Fluid

Order	Reagents	Amount in 1000 ml
1	NaCl	8.035 g
2	NaHCO_3_	0.355 g
3	KCl	0.225 g
4	K_2_HPO_4_.3H_2_O	0.231 g
5	MgCl_2_.6H_2_O	0.311 g
6	1.0 M HCl	39 ml
7	CaCl_2_	0.292 g
8	Na_2_SO_4_	0.072 g
9	Tris ((HOCH_2_)_3_CNH_2_)	6.118 g
10	1.0 M HCl	0–5 ml (*Appropriate amount* *for adjusting the pH 7.4)*

### SEM-EDX analysis

After 21 days, the samples were removed from the SBF. Samples were mounted on a glass slide using adhesive tape, placed upside down, and embedded in polyester resin. The hard wax in the cavities was removed. To perform the SEM-EDX analysis, the specimens were divided in the buccolingual direction, through the centers of the restorations, by using a water-cooled diamond blade (Fig. 1I) to expose the restoration–dentin interface. Next, the surfaces were sanded using 800–2000 grit sandpaper for 60 s. The samples were rinsed in an ultrasonic bath for 1 min to remove the residue and debris. Subsequently, they were air dried and coated with a 100-nm-thick gold/palladium layer.

**Fig. 3:**
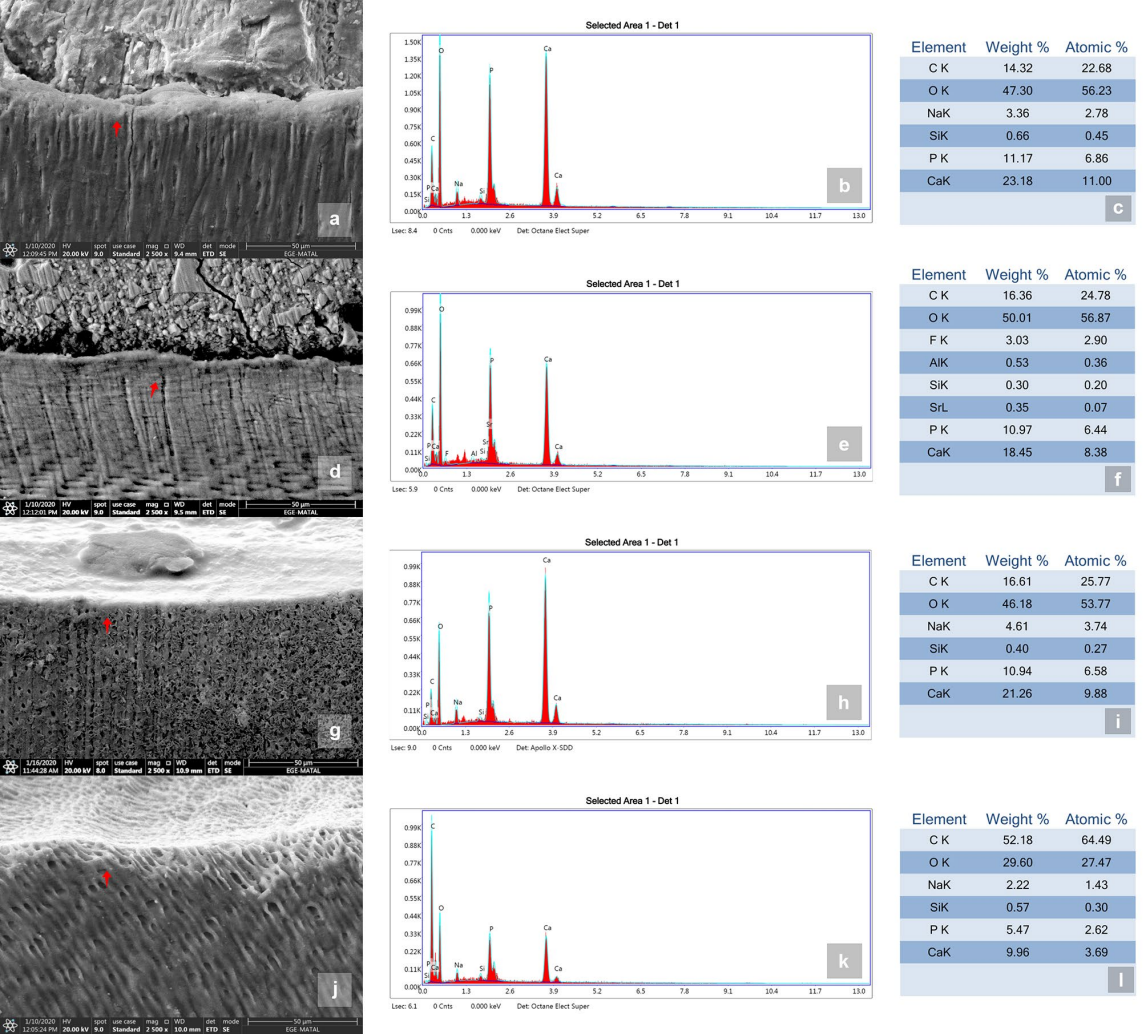
An example of scanning electron microscopy–energy-dispersive X-ray spectroscopy (SEM-EDX) analysis for each group. (**a–c**) Mapping images and elemental analysis of the Biodentine; (**d–f**) glass ionomer cement (GIC); (**g–i**) sound dentin as the positive control; (**j–l**) demineralized dentin as negative control. The elemental analysis shown on micrographs and tables was performed in selected regions (arrows)

SEM-EDX (Apreo™ 2 SEM for Materials Science, Thermo Fisher Scientific Inc., Waltham, Massachusetts, USA) was used to determine the ion transfer and the elemental content of the dentin in each group. EDX quantitative chemical analysis (% weight) and elemental mapping were performed to determine the Ca/P ratios (Fig. 3). To obtain elemental data, the samples were observed at × 2500 magnification at 20 kV. Multiple measurements of the dentin surfaces were obtained to minimize measurement errors, starting at the restoration–dentin interface and progressing at 20-µm intervals in the underlying sound dentin up to 100 μm. The average Ca/P ratio was calculated for each group using the data obtained.

### Statistical analysis

In this study, the Ca/P ratios determined by SEM-EDX analysis were used to compare remineralization between the four groups.

As the first step of the statistical analysis, the normality of the data distribution was checked using the Shapiro–Wilk test, and the homogeneity of variance was assessed using the Levene test. To examine the differences between the means of multiple dependent groups, a repeated-measures analysis of variance (ANOVA) test, which included the Greenhouse–Geisser correction, was conducted after confirming that the data were normally distributed. Post-hoc Bonferroni tests were used to determine the groups or subgroups that caused these differences (*p* < 0.05). All statistical analyses were performed using IBM SPSS, version 25.0 (SPSS Statistics, Chicago, IL, USA).

## Results

### Ca/P ratios of the samples

The Shapiro–Wilk analysis (Table 2) revealed that the Ca/P ratios of all four materials were normally distributed (all *p* > 0.05). Table 3 shows the mean, standard deviation, and minimum and maximum Ca/P ratios for each group, along with pairwise comparisons. Repeated-measures ANOVA and Bonferroni post hoc tests revealed that the Ca/P ratio of Group 2 (Biodentine) was significantly greater than that of all other groups (*p* < 0.001). Statistically significant differences were determined among the positive control group (Group 1), the Biodentine group (Group 2), the GIC group (Group 3), and the negative control group (Group 4); between the negative control and Biodentine, as well as the negative control and the GIC groups; and between the Biodentine and GIC groups (all *p* < 0.001).

**Table 2:** Normality of distribution according to study groups

	Shapiro–Wilk test statistics	*p* value
Positive control	0.943	0.075
Negative control	0.983	0.849
Biodentine	0.977	0.692
GIC	0.956	0.180

GIC, glass ionomer cement

**Table 3:** A pairwise comparison of the Ca/P ratio between the groups

	Min.	Max.	Mean	SD	F	*p* value	Bonferroni test results
Positive Control (Gr 1)	1.20	2.44	1.93	0.30	143.86	0.001*	Gr 1- Gr 2, 3, 4
Biodentine (Gr 2)	1.05	3.53	2.22	0.50			Gr 2 - Gr 3
GIC (Gr 3)	0.68	2.46	1.81	0.40			
Negative Control (Gr 4)	0.66	2.64	1.72	0.40			Gr 4 - Gr 2, 3

*n* = 34 in all groups. Gr, group

*p < 0.05

“-” indicates a significant difference

Data are presented as minimum, maximum, mean, and standard deviation

### SEM evaluation

**Fig. 4:**
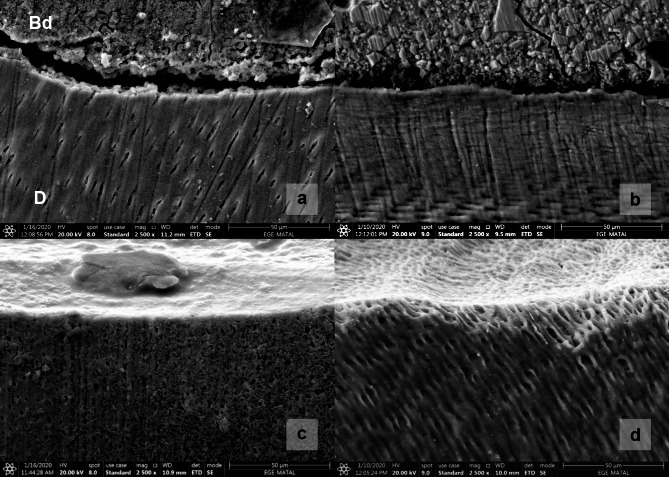
(**a**) The globular structure of Biodentine (Bd: Biodentine, d: dentin). (**b**) Glass ionomer cement, and the aluminum particles embedded in the matrix. (**c**) Sound dentin, as the positive control group, and the hydroxyapatite crystals around the dentin tubules. (**d**) Demineralized dentin, as the negative control group, where dentin tubules are observed to be open

**Fig. 5:**
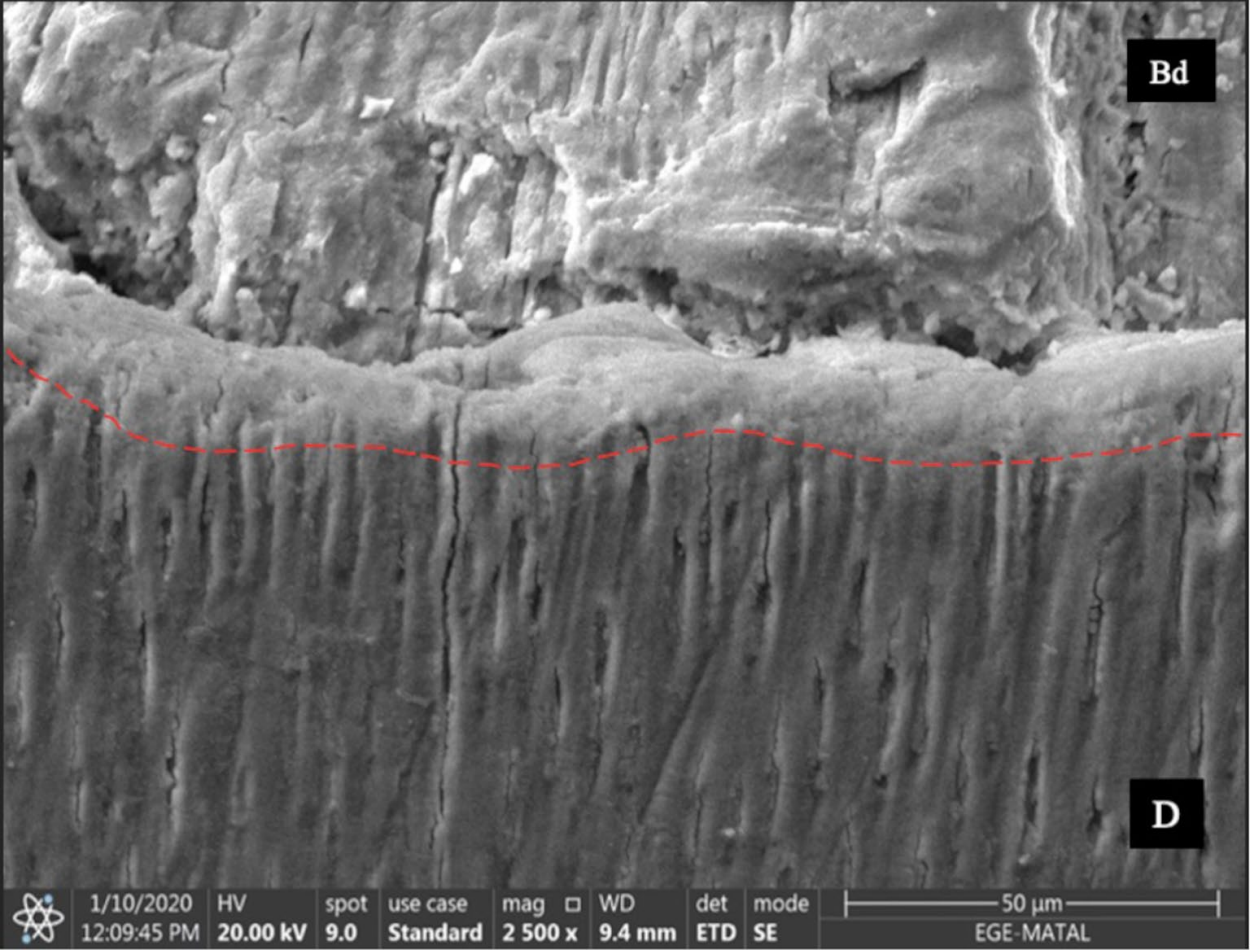
A remineralization layer in the dentin adjacent to the Biodentine in which tubules are observed to be occluded (Bd, Biodentine; D, dentin)

Biodentine had an irregular globular structure in the SEM images (Fig. 4A), whereas GIC contained aluminum particles embedded in the matrix (Fig. 4B). Group 1 showed hydroxyapatite crystals around the dentinal tubules (Fig. 4C). The dentinal tubules in Group 4 were open (Fig. 4D). In addition, SEM revealed a remineralized dentin layer, in which the tubules were occluded, adjacent to the Biodentine in Group 2 (Fig. 5).

## Discussion

In this study, we compared the remineralization capabilities of Biodentine and GIC (Fuji IX) on artificially induced carious dentin lesions by measuring the Ca/P ratio using SEM-EDX analysis. Our results demonstrated that Biodentine showed a significantly higher remineralization ability than that of GIC in artificial dentin lesions. Therefore, the null hypothesis was rejected.

There are two possible explanations for this result: First, Biodentine exhibits a caustic effect on dentinal collagen due to its high alkalinity and stimulates the denaturation of intertubular dentinal collagen fibrils, thereby creating a porous structure [22]. The space remaining after degradation is occupied by apatite-like minerals that penetrate the dentin tubules. Second, Biodentine powder contains calcium carbonate as a filler, which functions as a nucleation site by leaching Ca^2+^ and OH^−^ ions into the solution, allowing *de novo* nucleation of minerals in the presence of phosphate ions inside the dentin matrix [22, 33, 34].

In contrast, GIC is acidic and requires preexisting nucleation sites to induce remineralization [35]. The acidic effect enhances the water permeability and porosity of the peritubular dentin, which results in ion exchange between the GIC and the wet dentin underneath [18, 36]. These mechanisms involve the diffusion of calcium/strontium ions into the hypomineralized matrix, along with polyalkenoic acids that cause additional demineralization [36], resulting in the diffusion and *epitaxial growth* of the remaining crystals. Another key point is that GIC (Fuji IX) can release fluoride and strontium ions [18], which might have reduced the Ca and P (% w) concentrations in our analysis. According to a previous study, GICs do not produce hydroxyapatite crystals, as their acidic nature prohibits the production of apatite, which is contrary to the concept of bioactivity [37]. Our study revealed a significant difference in the Ca/P ratios between the groups, with Group 3 (GIC) exhibiting a significantly lower Ca/P ratio than that of Group 2 (Biodentine). This discrepancy aligns with the mechanisms discussed earlier, highlighting the superior remineralization capacity of Biodentine due to its alkaline nature and calcium carbonate filler.

In this study, an interactive interface layer in which the tubules were occluded was observed between the Biodentine and dentin in the SEM images. This layer was interpreted as the zone of intratubular mineralization. The results of this study were in accordance with those of previous studies that identified a similar remineralization layer [22, 38, 39]. According to Atmeh et al., this layer is mediated by the cement’s alkaline caustic effect on the organic component of dentin, which facilitates mineral transfer, resulting in the formation of a “mineral infiltration zone” (MIZ) [40]. In contrast, several studies have reported that ion transfer alone cannot explain the intrafibrillar remineralization of dentin [35, 41]. Because of its mineral-rich structure, we believe that the MIZ can reduce microleakage and improve remineralization.

In previous studies, the remineralization capacity of Biodentine and GIC has been investigated using various methodologies. Using two-photon fluorescence microscopy and Raman spectroscopy analysis to assess the remineralization of demineralized dentin, Sajini et al. demonstrated that Biodentine had remineralizing potential comparable to that of GIC, in addition to inducing remineralization of CAD [42]. Nunez et al. compared the effects of bioactive-glass-added-Biodentine and GIC using SEM-EDX analysis and reported that the Ca/P ratio of Biodentine was significantly higher than that of GIC [43]. Pires et al. created artificial dentin caries using a bacterial model and evaluated the remineralization potential of mineral trioxide aggregate, poly zinc cement, Biodentine, and GIC using micro-computed tomography (micro-CT). Their findings revealed that Biodentine had a higher remineralization ability than that of GIC [29]. Our findings were consistent with those of these previous studies and support the higher remineralization capacity of Biodentine.

Neves et al. compared the remineralization abilities of mineral trioxide aggregate, Portland cement, Biodentine, and GIC on bacterially-induced artificial dentin caries in bovine teeth, using micro-CT [44]. In contrast to the results of the present study, they reported that GIC had a greater capacity for remineralization than did Biodentine [44]. Variations in experimental designs and analyses could lead to differing outcomes. The remineralization process in GIC is primarily attributed to the release of fluoride and strontium ions [18]. Micro-CT measures remineralization by determining radiological density, rather than by analyzing the elemental composition. Consequently, remineralization regions exhibit a higher density when observed using micro-CT imaging [45]. However, in this study, the Ca/P ratio was calculated by using SEM-EDX to evaluate remineralization. Hence, the influence of fluoride and strontium ions on the Ca/P ratio was anticipated to be negligible.

Measuring mineral changes, determining their location, and identifying the responsible ions is important for identifying remineralization in dentin [46]. Various methods, each with its own advantages and limitations, are available for detecting remineralization in dentin. For instance, non-destructive techniques, such as micro-CT and two-photon fluorescence microscopy are useful for assessing remineralization dynamics; however, they do not provide information on apatite formation or changes in elemental composition. Micro-CT allows a three-dimensional evaluation of the sample with volumetric results, whereas Raman spectroscopy evaluates remineralization based on changes in the optical properties of the sample [42, 47]. In our study, we used SEM-EDX to assess remineralization by observing the dentin–restoration interface and evaluating ion changes in the dentin structure; however, this technique requires invasive sample preparation and additional caution to prevent changes in the natural condition of the specimen structure [4, 5]. Notably, because no gold standard for this assessment exists, the integration of diverse analytical tools has the advantage of mitigating technique-specific variations. This complementary approach serves to avoid potential biases and to increase the precision of the results.

In this study, an in vitro model was used to address the challenges posed by the heterogeneity in the natural structure, the variations in the extent of the caries lesion, and the lack of surface standardization in the dentin–restoration interface in clinical trials [23]. In addition, we used an acetic acid solution to create artificial caries, which resemble residual caries after selective caries removal. Acetic acid-induced lesions, without complete demineralization of the sample, resemble dentin caries [31]. In this study, Group 4, which had unrestored carious dentin, showed a significantly lower Ca/P ratio than that of the other groups. These results indicate that artificial caries induction is an effective means of mimicking residual dentin caries.

SBF was used as the remineralization medium to observe apatite formation and evaluate bioactivity. When a sample is immersed in SBF, calcium ions react with the phosphate groups of SBF, resulting in calcium phosphate precipitation. This process facilitates the nucleation and crystallization of hydroxyapatite on the sample surface. The gradual release of calcium from the bioactive material and the interaction of these ions with phosphorus compounds in the SBF causes remineralization at the restoration–dentin interface [48]. SBF is widely used in remineralization studies involving hard tissues [38, 49–52]. The in vitro apatite-forming ability, measured using the SBF test, has been assumed to predict in vivo bioactivity [53]. Moreover, Jang et al. reported that phosphate-buffered saline solution and SBF were equally effective for dentin remineralization [54]. In this study, we used SBF as a saliva substitute to provide phosphate, which is widely recognized for its role in biomineralization.

This study had several limitations. First, the caries-inducing and remineralization environments were completely chemical, and the potential inhibitory effects of an acidic bacterial environment on the observed mechanism were not considered. Second, the mineral change was assessed only on one surface using SEM-EDX. Although SEM-EDX allowed for detailed detection of elemental changes, it does not provide a complete picture of mineral density and lesion depth. Structural changes in the collagen matrix should be investigated, and data should be obtained from different depths and verified using different analysis tools such as Micro-CT or Transverse Microradiography. Future studies combining in-vitro and in-vivo approaches with different analysis tools are needed to achieve a comprehensive understanding of the mechanisms and their clinical applications.

## Conclusion

In conclusion, this study showed that both CSC (Biodentine™) and GIC (Fuji IX) induced remineralization in artificial dentin caries. However, Biodentine demonstrated a higher remineralization capacity than that of GIC. Biodentine may offer enhanced bioactivity, providing an effective seal and remineralization of carious dentin, due to its ability to form a mineral-rich interfacial layer and to accelerate apatite formation. Using Biodentine as a bioactive dentin restorative material will allow clinicians to treat deep dentin caries by using a minimally invasive approach to rehabilitate the tooth structure and preserve tooth vitality.

## Data Availability

The data supporting the conclusions of this article is included within the article. The dataset is available from the corresponding author upon reasonable request.
